# Clinical efficacy of THA with dual mobility cup vs. hemiarthroplasty in elderly patients with femoral neck fracture: a retrospective study

**DOI:** 10.3389/fsurg.2025.1507068

**Published:** 2025-02-05

**Authors:** Kai Xiao, Songyang Liu, Boran Liang, Shuming Li, Xinyan Liu, Jing Chen

**Affiliations:** ^1^ Department of Orthopedics, Aerospace Center Hospital, Beijing, China; ^2^ Beijing University of Posts and Telecommunications Library, Beijing, China

**Keywords:** femoral neck fracture, total hip arthroplasty, hemiarthroplasty, dual mobility cup, elderly patients

## Abstract

**Objective:**

To compare the clinical efficacy of total hip arthroplasty (THA) with or without dual mobility cup (DMC) vs. hemiarthroplasty (HA) in elderly Asian patients with acute femoral neck fracture (FNF).

**Methods:**

Data of 284 elderly FNF patients treated at our institution from January 2017 to December 2021 were retrospectively collected. Patients were divided into the DMC-THA group (THA with DMC, *n* = 102), C-THA group (conventional THA without DMC, *n* = 88), and HA group (*n* = 94). The study assessed perioperative outcomes, hip functional recovery, treatment satisfaction, long-term prognosis, and quality of life.

**Results:**

The Harris Hip Scores in the DMC-THA group were significantly higher than those in the C-THA and HA groups at 3 months, 6 months, and 1 year postoperatively (*P* < 0.05). The satisfaction rate in the DMC-THA group (92.2%) was significantly higher compared to the C-THA (81.8%) and HA groups (80.9%) (*P* < 0.05). At 1 year post-surgery, the DMC-THA group demonstrated a significantly lower dislocation rate (2.0% vs. 9.1%) and superior mobility compared to the C-THA group (*P* < 0.05). Additionally, the DMC-THA group exhibited significantly better mobility and reduced pain/discomfort compared to the HA group (*P* < 0.05).

**Conclusion:**

THA with DMC offers superior joint function recovery, a lower dislocation rate, and improved quality of life compared to conventional THA and HA, positioning it as a preferred surgical option for elderly patients with acute FNF.

## Introduction

Femoral neck fracture (FNF) is one of the most common fracture types, mostly occurring in the elderly, which is closely related to osteoporosis, hip muscle loss, poor stability, easy to fall and other factors (1). FNF can easily damage the surrounding blood supply vessels, thus leading to fracture nonunion or ischemic necrosis of the femoral head. Moreover, given multiple comorbidities and diminished physiological reserve, prolonged conservative management frequently leads to complications like pressure ulcers and hypostatic pneumonia (2, 3). Early surgical intervention is therefore recommended to enable early mobilization, enhance functional recovery, and reduce mortality in elderly FNF patients.

Total hip arthroplasty (THA) and hemiarthroplasty (HA) are two common surgical options for FNF. While THA offers superior hip functional rehabilitation and diminished hip pain when contrasted with HA (4, 5), its drawback lies in an elevated dislocation incidence (5, 6), adversely affecting its overall therapeutic efficacy. The development of the dual mobility cup (DMC) was conceived as a countermeasure to mitigate the dislocation vulnerability associated with conventional THA (7). The objective of this study was to compare the clinical efficacy of THA with DMC vs. conventional DMC and HA in the treatment of FNF, and to explore the most appropriate treatment for elderly population.

## Methods

### Inclusion/exclusion criteria and sample size

A total of 284 elderly patients with acute FNF admitted to our institution from January 2017 to December 2021 were included. Inclusion criteria: (1) Age over 60 years. (2) Preoperative X-ray and CT examination confirmed Garden III-IV unilateral FNF. (3) Able to walk wityout any assistive devices before injury. (4) ASA grade I-III. (5) Informed consent. Exclusion criteria: (1) Presence of psychiatric or neurological diseases. (2) Old fracture, multiple fracture, or pathological fracture. (3) Complicated with severe vascular and nerve injury. (4) History of previous operations in the affected limb. This study met the relevant requirements of the Helsinki Declaration, and approved by the Ethics Committee of our institution. According to different surgical methods, patients were then divided into DMC-THA group (*n* = 102), C-THA group (*n* = 88), and HA group (*n* = 94).

In this retrospective study, we estimated the sample size based on power analysis to ensure adequate statistical power to detect clinically meaningful differences between groups. We used SPSS to calculate the required sample size, setting the significance level (*α*) at 0.05 and the statistical power at 0.80 (80%). The effect size was determined based on previously reported studies, with a minimum detectable difference of Harris scores set at 3.2 points.The estimated sample size was 86 in each group.We aimed to include the maximum available sample size from the eligible records within the study period, recognizing that the effective sample size could be smaller than initially estimated. Given the retrospective nature of the study, we were limited to the number of patients who met the inclusion criteria during the study period.

### Characteristics of the patients

In the DMC-THA group, there were 43 males and 59 females, with an average age of (68.7 ± 4.6) years. 58 fractures were on the left side and 44 fractures on the right side. The causes of injury were as follows: traffic accidents in 22 cases, slip down in 58 cases and falls in 32 cases. In the C-THA group, 39 cases were male and 49 cases were female, with an average age of (69.6 ± 5.1) years. There were 48 cases of left fractures and 40 cases of right fractures. The causes of injury were as follows: 18 cases of traffic accident, 44 cases of slip down and 26 cases of fall. In the HA group, there were 42 males and 52 females, with an average age of (69.1 ± 5.3) years, 52 fractures on the left side and 42 fractures on the right side. The causes of injury were as follows: traffic accidents in 19 cases, slip down in 50 cases and falls in 25 cases. The general clinical data of the three groups were similar, and the differences were not statistically significant (*P* > 0.05, Table 1).

**Table 1:** Demographic and relevant clinical data of the study population.

Variable	DMC-THA *n* = 102	C-THA *n* = 88	HA *n* = 94	*P*
Gender, *n* (%)				
Male	43 (42.2)	39 (44.3)	42 (44.7)	0.565
Female	59 (57.8)	49 (55.7)	52 (55.3)	0.61
Mean age ± SD(yrs)	68.7 ± 4.6	69.6 ± 5.1	69.1 ± 5.3	0.113
Fracture side, *n* (%)				
Left side	58 (56.9)	48 (54.5)	52 (55.3)	0.257
Right side	44 (43.1)	40 (45.5)	42 (44.7)	0.291
BMI, *n* (%)				
<30	72 (70.6)	64 (72.7)	70 (74.5)	0.346
30–40	22 (21.6)	18 (20.5)	19 (20.2)	0.394
>40	8 (7.8)	6 (6.8)	5 (5.3)	0.657

### Treatment

After successful anesthesia, the patient was placed in a healthy lateral decubitus position, and the surgical area was routinely disinfected and draped. The joint capsule was dissected to expose the neck of the femur. The femoral neck osteotomy was performed and the diameter of the femoral head was measured.

#### THA

The soft tissue and osteophytes in the acetabulum labrum and fossa were cleared, and the acetabulum cartilage surface was filed with a small acetabulum and polished until the bottom of the acetabulum was exposed, and then polished to the subchondral bone with a diameter less than 2 cm of the femoral head in the direction of 45° and 15° anteriorly, and an appropriate acetabulum cup was selected for placement and compression fixation. Hip flexion, bend the knee, turn the hip direction and expose the proximal end of the femur. Gradually reaming the pulp with a medullary file and placing the femoral side prosthesis at a 10–15° forward angle. The hip joint is reduced by pressing the femoral head into the lining with an indenter. The hip joint is then moved to check the stability. After excluding the active bleeding, drainage was placed and the incisions were closed layer by layer. During the operation, DMC-THA group and C-THA group used DMC and conventional total hip prosthesis, respectively.

#### HA

The head ligaments in the coabulum equine fossae were cleaned, and the labrum and joint capsule of the acetabulum were retained. Hip flexion, bend the knee, shift the direction of the hip joint and expose the proximal end of the femur. A suitable artificial femoral head prosthesis was selected, and the model was placed according to the intertrochanteric line plane. The size and tightness of the model were checked. The prosthesis was implanted and the hip joint was reduced, and then the hip joint was moved to check the stability. After excluding the active bleeding, drainage was placed and the incisions were closed layer by layer.

Antibiotics were routinely used 30 min before surgery and 48 h after surgery to prevent infection. Patients in HA group were placed in abduction neutral position after surgery, while patients in the DMC-THA and C-THA groups had no special postural requirements. Heparin sodium and lower extremity elastic socks were routinely used to prevent thrombosis. The lower limb muscle exercise was started on the 1st day after surgery, and the drainage condition was observed. If there was no abnormality, the drainage was removed on the 2nd day after surgery. The patients walked with assistance at 2–4 days after surgery, walked with full weight at 2–4 weeks after surgery, and began to walk independently without a walker within 1 month after surgery.

### Outcome measures

(1) Perioperative outcomes: including operation time, intraoperative blood loss, postoperative drainage volume, perioperative blood transfusion, length of getting out of bed, length of hospital stay and death. (2) Recovery of hip function: the Harris score (8) was used to evaluate hip function before surgery, 3 months, 6 months, and 1 year after surgery. The Harris score is out of 100, with higher scores indicating better hip function. (3) Treatment satisfaction rate: All patients were evaluated 3 months after surgery and divided into four dimensions: excellent, good, fair, and poor. Treatment satisfaction rate was defined as the proportion of patients rated as excellent and good in the whole group. (4) Long-term prognosis: including dislocation, reoperation, and death. (5) Quality of life: The European Quality of Life (EQ-5D) score (9) was used to assess quality of life at 1 year after surgery. The EQ-5D score consists of five dimensions: mobility, self-care, daily activities, pain/discomfort, and anxiety/depression, with three levels for each dimension.

### Statistical analysis

The SPSS 23.0 software was used for statistical analysis. Count data were presented as *n* (%), and differences between groups were assessed using the *χ*^2^ test or Fisher exact test. Quantitative data were expressed as (*x* ± *s*), and inter-group differences were assessed using the one-way ANOVA *P* < 0.05 indicates statistically significant. Post-hoc comparisons were conducted using Tukey's HSD test to assess pairwise differences between groups.

## Results

### Perioperative outcomes

Compared with the DMC-THA group, the HA group had a shorter operation time (76.8 ± 24.5 vs. 86.4 ± 26.6, *P* < 0.05) and less blood loss (257 ± 48 vs. 285 ± 53, *P* < 0.05). Postoperative drainage, perioperative blood transfusion, time to ambulation, and length of stay in the two groups were similar (*P* > 0.05). The perioperative treatment conditions between the DMC-THA and C-THA groups were similar, and the difference was not statistically significant (*P* > 0.05, Table 2). None of the three groups died during hospitalization or within 1 month after surgery.

**Table 2:** Comparison of the perioperative outcomes among the three groups.

Group	Operation time (min)	Blood loss (ml)	Postoperative drainage (ml)	Blood transfusion	Time to ambulation	Length of stay
DMC-THA group (*n* = 102)	86.4 ± 26.6	285 ± 53	128 ± 24	11 (10.8%)	3.9 ± 1.3	11.4 ± 3.1
C-THA group (*n* = 88)	88.2 ± 24.1	296 ± 60	131 ± 25	10 (11.4%)	4.2 ± 1.4	11.8 ± 3.4
P1 value	0.628	0.181	0.400	0.899	0.128	0.398
HA group (*n* = 94)	76.8 ± 24.5	257 ± 48	122 ± 20	9 (9.6%)	4.2 ± 1.1	11.7 ± 2.9
P2 value	0.009	<0.001	0.060	0.780	0.084	0.486

P1: The difference between DMC-THA and C-THA group.

P2: The difference between DMC-THA and HA group.

### Recovery of hip function

Preoperative Harris scores among the three groups were similar (*P* > 0.05). Postoperative Harris scores of the three groups were significantly higher than those before surgery, and gradually increased with the extension of time. At 3 months, 6 months and 1 year after surgery, the Harris scores of the DMC-THA group were significantly higher than those of the C-THA and HA group (*P* < 0.05, Table 3).

**Table 3:** Comparison of the recovery of hip function among the three groups.

Group	Preoperative	3 m after surgery	6 m after surgery	1 y after surgery
DMC-THA group (*n* = 102)	24.7 ± 6.1	76.6 ± 8.7	84.2 ± 10.0	89.5 ± 9.1
C-THA group (*n* = 88)	23.9 ± 5.4	72.3 ± 10.3	80.6 ± 8.8	86.2 ± 9.7
P1 value	0.343	0.002	0.010	0.017
HA group (*n* = 94)	24.3 ± 5.3	70.1 ± 8.5	80.4 ± 11.3	85.0 ± 10.2
P2 value	0.626	<0.001	0.013	0.001

P1: The difference between DMC-THA and C-THA group.

P2: The difference between DMC-THA and HA group.

### Treatment satisfaction

The patients' satisfaction with treatment was investigated 3 months after operation, and the follow-up rate was 100%. The treatment satisfaction rate of DMC-THA group (92.2%) was significantly higher than those of the C-THA (81.8%) and HA groups (80.9%, *P* < 0.05). See Table 4.

**Table 4:** Comparison of the treatment satisfaction among the three groups.

Group	Excellent	Good	Fair	Poor	Satisfaction rate
DMC-THA group (*n* = 102)	22 (21.6%)	72 (70.6%)	7 (6.9%)	1 (1.0%)	94 (92.2%)
C-THA group (*n* = 88)	16 (18.2%)	56 (63.6%)	14 (15.9%)	2 (2.3%)	72 (81.8%)
*χ* ^2^	–	–	–	–	4.576
P1 value	–	–	–	–	0.032
HA group (*n* = 94)	20 (21.3%)	56 (59.6%)	16 (17.0%)	2 (2.1%)	76 (80.9%)
χ^2^	–	–	–	–	5.435
P2 value	–	–	–	–	0.020

P1: The difference between DMC-THA and C-THA group.

P2: The difference between DMC-THA and HA group.

### Long-term prognosis

All patients completed 1-year postoperative follow-up. Compared with the C-THA group, the dislocation rate in the DMC-THA group was significantly lower (2.0% vs. 9.1%, *P* < 0.05). The dislocation rate was similar between the DMC-THA and HA groups (*P* > 0.05). In addition, the reoperation rate and 1-year mortality of the three groups were similar, and the differences were not statistically significant (*P* > 0.05, Table 5).

**Table 5:** Comparison of the long-term prognosis among the three groups.

Group	Dislocation	Reoperation	Death
DMC-THA group (*n* = 102)	2 (2.0%)	0 (0.0%)	4 (2.9%)
C-THA group (*n* = 88)	8 (9.1%)	2 (2.3%)	5 (5.7%)
P1 value	0.046	0.213	1.000
HA group (*n* = 94)	4 (4.3%)	1 (1.1%)	4 (4.3%)
P2 value	0.429	0.480	0.735

P1: The difference between DMC-THA and C-THA group.

P2: The difference between DMC-THA and HA group.

### Quality of life

**Figure 1:**
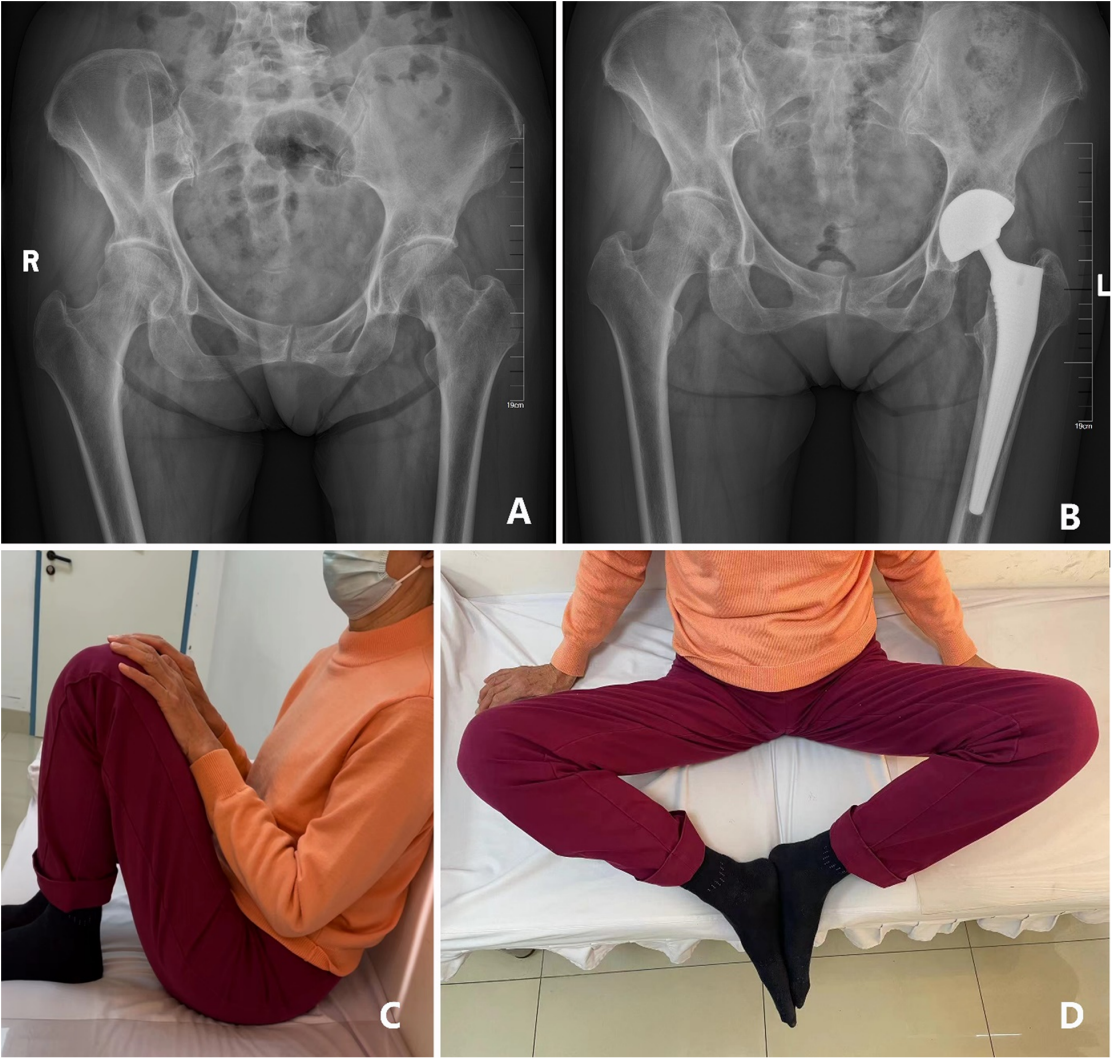
**(A)** the pelvic radiograph shows a left femoral neck fracture in a 72 years old female patient who experienced a fall; **(B)** A satisfied radiographic outcome was observed after 3 years follow-up following DMC-THA; **(C,D)** the patient achieved execellent hip function at 3 years follow-up.

Except for patients who died within 1 year after surgery, all patients completed the EQ-5D quality of life questionnaire at 1 year after surgery. Compared with the C-THA group, the mobility of the DMC-THA group was significantly better (*P* < 0.05) (Figure 1). Compared with the HA group, the mobility and pain/discomfort in the DMC-THA group were significantly better (*P* < 0.05). There were no statistically significant differences in self-care ability, daily activities and anxiety/depression among the three groups (*P* > 0.05). See Table 6.

**Table 6:** Comparison of the quality of life among the three groups.

Group	Mobility			Self-care			Daily activities		
	No problems	Some problems	A lot of problems	No problems	Some problems	A lot of problems	No problems	Some problems	A lot of problems
DMC-THA group (*n* = 102)	71 (69.6%)	31 (30.4%)	0 (0.0%)	61 (59.8%)	38 (37.3%)	3 (2.9%)	58 (56.9%)	38 (37.3%)	6 (5.8%)
C-THA group (*n* = 88)	49 (55.7%)	39 (44.3%)	0 (0.0%)	43 (48.9%)	42 (47.7%)	3 (3.4%)	38 (43.1%)	43 (48.9%)	7 (8.0%)
χ^2^	4.410			2.702			4.431		
P1 value	0.036			0.259			0.109		
HA group (*n* = 94)	48 (51.1%)	46 (48.9%)	0 (0.0%)	42 (44.7%)	49 (52.1%)	3 (3.2%)	44 (46.8%)	44 (46.8%)	6 (6.4%)
χ^2^	6.566			4.164			2.403		
P2 value	0.010			0.125			0.301		

Group	Pain/discomfort			Anxiety/depression		
	No	Some	A lot	No	Some	A lot
DMC-THA group (*n* = 102)	63 (61.8%)	37 (36.3%)	2 (1.9%)	77 (75.5%)	23 (22.5%)	2 (2.0%)
C-THA group (*n* = 88)	47 (53.4%)	37 (42.0%)	4 (4.6%)	63 (72.3%)	24 (26.5%)	2 (1.2%)
χ^2^	1.122			0.557		
P1 value	0.571			0.757		
HA group (*n* = 94)	42 (44.7%)	45 (47.9%)	7 (7.4%)	64 (68.1%)	26 (27.7%)	4 (4.2%)
χ^2^	7.070			1.475		
P2 value	0.029			0.478		

P1: The difference between DMC-THA and C-TH A group.

P2: The difference between DMC-THA and HA group.

## Discussion

With the escalating proportion of the elderly population in Asian, the incidence of FNF is progressively rising. Regarding the treatment, consensus holds that surgical treatment, if feasible, is necessary to achieve better clinical outcome (10, 11). However, there is still some controversy over which surgical method should be selected (12). Compared with THA, HA offers the advantages of short operation time, procedural simplicity and less surgical blood loss, which was supported by our study. However, HA was not superior to THA in postoperative hip functional recovery. Therefore, previous studies has commonly recommended elderly patients with advanced age, weak physical condition and poor pre-injury activity to receive HA (12–14).

Dislocation of joint prostheses is a common complication following THA, proportionally escalating with *in vivo* use, with a long-term dislocation rate of more than 10% (15). Factors affecting dislocation include prosthesis placement, surgical approach, soft tissue management around hip joint and other surgical factors, along with patient factors such as previous hip surgery history, neuromuscular disorders and postoperative rehabilitation exercises (16). In order to address the shortcoming of THA, Professor G.Bousquet proposed the concept of DMC system in the mid-1970s. Comprising a metal outer shell and a freely moving polyethylene insert, the DMC features two interfaces for motion—a small interface between the ball head and polyethylene insert, and a larger interface between the polyethylene insert and the metal shell. Both interfaces share a common center of motion. The polyethylene insert allows flexible ball head rotation with minimal friction, effectively fulfilling most hip joint functions. Additionally, the insert achieves “locking” through a pressure mechanism, combining freedom of movement with secure integration. During the joint intervention of the large joint motion interface in the hip joint extreme movement, the insert rotates freely in the metal cup and actively capture the ball head, enhancing joint stability and yielding an initial THA dislocation rate of merely 0%–1.1% (17).

Dual-mobility THA generally achieves lower dislocation rates compared to traditional THA and HA. Studies have indicated that DM designs offer greater stability, with some showing a zero-dislocation rate for DM cups in both primary and revision surgeries for high-risk patients (e.g., elderly or those with FNF) (18). This is a major benefit over traditional THA, where instability often remains a leading cause for revision (19). Studies also indicate that DM THA offers better overall stability than hemiarthroplasty for patients undergoing conversion surgeries after failed HA. In cases of recurrent dislocations with HA, DM constructs effectively reduce instability without significantly increasing mortality risks in older or frail patients (20).The results of our study echoed those researches (21), with the dislocation rate of the DMC-THA group was significantly lower than that of the C-THA group. Given the Asian population's typically shorter stature, particularly among women in the 150–155 cm range, acetabular size tends to be smaller, necessitating usage of smaller ball heads during surgery. This elevates the risk of reduced hip joint range of motion and subsequent dislocation. In this term, DMC is more suitable for patients with small acetabulum and has more obvious advantages in preventing dislocation.

In terms of patient outcomes, dual-mobility THA has been associated with significant improvements in functional scores, like the Harris Hip Score, which reflects better mobility and quality of life (22). Our results demonstrated that the Harris scores of each postoperative stage in the DMC-THA group were significantly higher than those in the C-THA and HA groups. This outcome aligns with the design strengths of the DMC. In theory, DMC can play the role of a large-diameter femoral head, which can affect the “jump distance” to improve the stability of the prosthesis. It also reduces the occurrence of hip impaction by modifying head-to-neck ratios (23), broadening the range of motion before impaction and dislocation and restoring the range of motion close to the physiological joint. The thickness of the metal acetabulum cup on the acetabulum side resembles the thickness of the cartilage of the osseous acetabulum, allowing for anatomical replacement. With the clever design of acetabulum bionics, the space size and position of the acetabulum are almost unchanged. The top of the biological pressure matching mortar cup is cut flat, which increases the insertion depth of the mortar cup. The annular groove design cup's surface can increase the contact area between the bone and the mortar cup and form good initial stability. This has also been verified in medium - and long-term follow-up studies, with the survival rate of the prosthesis as high as 96.7% (24). Interestingly, Andriollo et al. assessed the outcomes of uncemented long-stemmed bipolar hemiarthroplasty for treating unstable intertrochanteric fractures in elderly patients, focusing on functional recovery and complication rates. Results indicate that this procedure allows early mobility with a low complication rate, making it a suitable option for elderly patients with limited life expectancy and significant comorbidities (25).

The integration of dual-mobility (DM) total hip arthroplasty with postoperative rehabilitation and monitoring strategies shows significant potential for improving patient outcomes, though further exploration is warranted. Studies highlight the effectiveness of structured rehabilitation, especially home-based programs, in enhancing recovery post-THA. For instance, a resistance-band home-based program demonstrated improved mobility, functional capacity, and quality of life for THA patients, indicating a practical and cost-effective approach (26). Remote rehabilitation supported by devices like wearable sensors and tablet apps can facilitate ongoing recovery at home. Patients monitored through mobile devices for exercises and step count show improved adherence to rehabilitation protocols, with promising results for long-term mobility gains (27).

This study has several limitations that may affect the generalizability and robustness of its conclusions. First, while the results support the advantages of DMC-THA) over conventional THA and HA)in reducing dislocation rates and improving functional outcomes, the study's findings are primarily limited to an elderly Asian cohort. This may limit applicability to non-Asian populations with different anthropometric and lifestyle characteristics, which should be noted when considering the broader relevance of DMC-THA.Second, the retrospective design and sample size, while based on available cases meeting inclusion criteria, may not capture the full diversity of patient outcomes. Larger, prospective studies with broader inclusion criteria and randomized controls would strengthen the findings presented here. Additionally, certain factors such as patient selection and exclusion criteria were carefully defined to maintain study consistency, but they could introduce potential selection bias, which may influence the results. Furthermore, this study does not extensively address cultural, social, and healthcare system differences, which could influence patient preferences, postoperative care, and recovery outcomes. These factors should be considered in future studies aiming to evaluate the efficacy of DMC-THA in diverse healthcare environments.

In conclusion, THA combined with DMC can reduce the risk of prosthesis dislocation, improve the postoperative hip functional recovery, and improve the quality of life and treatment satisfaction, which should be recommended as the preferred replacement program for elderly Asian patients with acute FNF.

## Data Availability

The raw data supporting the conclusions of this article will be made available by the authors, without undue reservation.
